# Response to Treatment with TNFα Inhibitors in Rheumatoid Arthritis Is Associated with High Levels of GM-CSF and GM-CSF^+^ T Lymphocytes

**DOI:** 10.1007/s12016-017-8610-y

**Published:** 2017-05-09

**Authors:** Jonas Bystrom, Felix I. Clanchy, Taher E. Taher, Mohammed M. Al-Bogami, Hawzheen A. Muhammad, Saba Alzabin, Pamela Mangat, Ali S. Jawad, Richard O. Williams, Rizgar A. Mageed

**Affiliations:** 1 Centre for Experimental Medicine and Rheumatology, William Harvey Research Institute, Barts and the London School of Medicine and Dentistry, Queen Mary University of London, Charterhouse Square, London, EC1M 6BQ UK; 2 Kennedy Institute of Rheumatology, Oxford University, Oxford, UK; 3 Department of Rheumatology, Royal Free Hospital, NHS Fundation Trust London, London, UK

**Keywords:** TNFα, Rheumatoid arthritis, T lymphocytes, GM-CSF

## Abstract

Biologic TNFα inhibitors are a mainstay treatment option for patients with rheumatoid arthritis (RA) refractory to other treatment options. However, many patients either do not respond or relapse after initially responding to these agents. This study was carried out to identify biomarkers that can distinguish responder from non-responder patients before the initiation of treatment. The level of cytokines in plasma and those produced by ex vivo T cells, B cells and monocytes in 97 RA patients treated with biologic TNFα inhibitors was measured before treatment and after 1 and 3 months of treatment by multiplex analyses. The frequency of T cell subsets and intracellular cytokines were determined by flow cytometry. The results reveal that pre-treatment, T cells from patients who went on to respond to treatment with biologic anti-TNFα agents produced significantly more GM-CSF than non-responder patients. Furthermore, immune cells from responder patients produced higher levels of IL-1β, TNFα and IL-6. Cytokine profiling in the blood of patients confirmed the association between high levels of GM-CSF and responsiveness to biologic anti-TNFα agents. Thus, high blood levels of GM-CSF pre-treatment had a positive predictive value of 87.5% (61.6 to 98.5% at 95% CI) in treated RA patients. The study also shows that cells from most anti-TNFα responder patients in the current cohort produced higher levels of GM-CSF and TNFα pre-treatment than non-responder patients. Findings from the current study and our previous observations that non-responsiveness to anti-TNFα is associated with high IL-17 levels suggest that the disease in responder and non-responder RA patients is likely to be driven/sustained by different inflammatory pathways. The use of biomarker signatures of distinct pro-inflammatory pathways could lead to evidence-based prescription of the most appropriate biological therapies for different RA patients.

## Introduction

Rheumatoid arthritis (RA) is a debilitating disease characterised by autoimmunity and the production of high levels of pro-inflammatory cytokines resulting in joint inflammation, the production of matrix-degrading enzymes and cartilage and bone destruction [1]. Epidemiological and genetic studies indicate that susceptibility to RA is associated with HLA-DR alleles and genes encoding cytokines and proteins that regulate T cell responses, highlighting the importance of T cells in disease pathogenesis [2].

Knowledge of the principal pro-inflammatory mediators that cause synovial inflammation in RA led to the discovery and use of biological TNFα inhibitors as effective therapies for treating patients [3]. Most treated patients undergo remission, especially if anti-TNFα is administered soon after diagnosis [4, 5]. However, a significant proportion of patients established disease either do not respond to the treatment or relapse after an initial response, suggesting that the disease in these patients is not driven by TNFα. Exactly why some RA patients do not respond to TNFα inhibitors while others relapse after an initial response remains unclear. One possible explanation for the inconsistent or incomplete response of RA patients to anti-TNFα is that TNFα, perhaps paradoxically, also has regulatory functions. For example, although there is good evidence that the activity of regulatory T cells improves following treatment with anti-TNFα [6–8], there is also evidence that TNFα modulates TCR-mediated signalling [9, 10]. An alternative explanation may be that chronic inflammation and disease processes in non-responder patients are not wholly driven by TNFα. In this regard, a number of laboratories including ours have revealed that T cells from non-responder RA patients produce high levels of IL-17 and have a high frequency of circulating Th17 cells [11, 12]. Moreover, our studies have revealed that treating patients with TNFα inihibitors increases the frequency of Th17 cells and IL-17 production, possibly through increasing IL-12/IL-23 p40 production by monocytes/macrophages [12]. Although the molecular basis and the physiological and therapeutic implications remain to be determined, these findings indicate that TNFα/TNFα blockade have significant impacts on the function and frequency of T cell subsets [13]. This suggestion is consistent with the association between T cell responses and signalling generated by TNFα through its two receptors in T cells [reviewed in [14]].

The available evidence suggests that mechanisms driving inflammation in RA could be different in different patients as indicated by variations in the response of individual patients to treatment with anti-TNFα [7, 11, 12]. Therefore, a major challenge has been in the identification of alternative pathways that drive disease in non-responder patients before treatment with TNFα inhibitors starts to enable better, evidence-based therapeutic use of these biological agents. A large number of studies from many laboratories explored the predictive value of many biomarkers including cytokines produced by peripheral blood mononuclear cells or the transcriptome prior to treatment (summarised in Table 1). In the current study, we assess the phenotype and response of T cell subsets in RA patients prescribed anti-TNFα before treatment in relation to the patient’s subsequent response/lack of response to treatment. The results provide evidence for relationships between response/lack of response to anti-TNFα and the frequency of T cell subsets and patterns of plasma levels of pro-inflammatory cytokines prior to treatment.

**Table 1:** Reported studies identifying predictive biomarkers of RA patients’ responsiveness to TNFα inhibitors

Cytokines	Clinical sample	Protocol and outcome	Reference
Biomarkers studied			
12 cytokines/chemokines	Serum	Protein biochip array; high levels of MCP-1 and EGF associate with the response to etanercept	[15]
TNFα, IL-1β, IL-6	White blood cells (WBCs)	LPS-stimulated WBCs; high levels of IL-1β predicted responsiveness to TNFα inhibitors	[16]
IFNβ/IFNα	Serum	Functional reporter cell assay; increased ratio of IFNβ to IFNα strongly associated with non-response to TNFα inhibitors	[17]
Cytokines + autoantibodies	Serum	Antigen array and multiplex cytokine assay; identified multi-parameter proteins that predicted response to etanercept	[18]
Chemokines/inflammatory			
Mediators			
CXCL13	Plasma	ELISA; high baseline levels associated with remission at 2 years following treatment with TNFα inhibitors	[19]
CXCL10 + CXCL13	Serum	ELISA; elevated baseline levels were associated with favourable response to TNFα inhibitors	[20]
ICAM1 + CXCL13	Serum	Electrochemiluminescence; high soluble ICAM and low CXCL13 levels predict good response to anti-TNFα	[21]
MRP8/14	Serum	ELISA; high levels of the myeloid-related protein (MRP)8/14 complex (endogenous TLR-4 receptor agonist) predicted response to TNFα inhibitors	[22]
mRNAs			
Panel of 8 transcripts	Blood mononuclear cells (BMNCs)	Microarray and quantitative RT-PCR; transcripts for ribosomal components, cell adhesion and inhibition of migration/invasion, cytochromes, proteasome- mediated proteolysis, enzymes and signalling predicted responsiveness to infliximab	[23]
Panel of 8 transcripts	Whole blood	Microarray; transcripts of genes encoding HLA-DRB3, SH2D1B, GNLY, CAMP, SLC2A3 and IL2RB, MXD4 and TLR5 predicted response to infliximab	[24]
Panel of 8 transcripts	Whole blood	High-throughput RNA sequencing, DNA genotyping and proteomics; transcripts for SORBS3, AKAP9, CYP4F12, MUSTN, CX3CR1, SLC2A3, C21orf58 and TBC1D8 two SNPs and plasma sICAM1/CXCL13 protein ratio predicted responsiveness to TNFα inhibitors	[25]
Micro RNA 23 (miR-23) and miR-223	Serum	miRNA polymerase chain reaction (PCR) array; miR-23 predicted response to anti-TNFα/DMARDs combination therapy	[26]
miR-22 and miR-886.3p	Serum	Micro RNA array; low expression of miR-22 and high expression of miR-886.3p associated with good response to adalimumab and methotrexate.	[27]

## Patients and Methods

### Patients

Patients were recruited from rheumatology clinics at Barts Health NHS, the Royal Free London NHS Foundation and Imperial College Healthcare Trusts with patients’ informed consent. The study was approved by the Ethical Committee (06/Q0605/8; NRES Committee London—City & East) and conducted in compliance with the Declaration of Helsinki 2013. The patients were prescribed anti-TNFα based on NICE guidelines which include failure to respond to treatment with two disease-modifying agents one of which is methotrexate and with a DAS28 score >5.1. Patients were treated with adalimumab, certolizumab pegol, golimumab or etanercept in combination with methotrexate. Patients’ response to treatment was based on the European League Against Rheumatism (EULAR) response criteria [28]; patients were considered responders when their DAS28 decreased by >1.2 to ≤3.2 after 3 months of treatment. All other treated patients were considered non-responders. Immediately prior to treatment and after 1 and 3 months of treatment, 25 mL blood was drawn and B and T cells and monocytes enriched and phenotypically characterised by FACS stimulated and cultured. Plasma were separated and stored at −80 °C until tested.

### Reagents

PerCP-Cy5.5-conjugated anti-TNFα, eFluor® 660 anti-GM-CSF, PE-Cy7 anti-IFNγ, PE anti-IL-17A, FITC-anti-CD45RA, PE-anti-CD45RO, eFluor® 450-anti-CD161 and unconjugated anti-CD28 antibodies (Abs) were from eBioscience. Brilliant Violet 605-anti-CCR6 and APC-anti-IL-10 Abs were purchased from BioLegend. RosetteSep negative-enrichment kits for human T cells, B cells and monocytes were from StemCell Technologies. Ficoll-Paque was from GE Healthcare, RPMI from Lonza and foetal bovine serum from Life Technologies. Lipopolysaccharide (*E. coli*, serotype 0111:B4), ionomycin and PMA were from Sigma-Aldrich and Golgiplug™ was from BD Bioscience and Leucoperm from AbD Serotec.

### Cell Enrichment and Analysis

Blood T and B cells and monocytes were enriched by negative selection with RosetteSep kits using mononuclear cells separated by Ficoll-Paque centrifugation. The purity of enriched T cells was consistently ≥95% while for monocyte and B cells purity was 86–98%. The cells were suspended in RPMI containing 10% FCS and antibiotics and analysed by FACS or stimulated in vitro. T cells were stimulated with 10 μg/mL anti-CD3 mAb (clone OKT3) pre-coated onto sterile tissue culture plates and 10 μg/mL anti-CD28 (clone 28.2) in the medium. B cells were stimulated with 10 μg/mL goat F(ab’)_2_ anti-human IgM coated onto wells of the plates and 10 μg/mL antibody to CD40 (clone G28-5) in the medium. Monocytes were stimulated with 1 μg/mL LPS (*E. coli*, serotype 0111:B4). Supernatants were collected after 48 h, debris removed by centrifugation and cytokines quantified using Meso Scale Discovery (MSD) multiplex kits or ELISA. To verify that levels of TNFα produced by T cells and monocytes were representative of what the cells produce independent of the stimulation protocol, T cells and monocytes from three healthy controls (HCs) were also stimulated independently for 48 h with 0.1 μg/mL PMA and ionomycin. In addition, T cells were stimulated with 0.1 μg/mL PMA and ionomycin in the presence of 1 μl/mL Golgiplug™ (BD Bioscience) overnight to determine the level of intracellular cytokines. The cells were stained for CD45RA, CD45RO, CCR6 and CD161 by incubation with conjugated monoclonal antibodies (mAbs) for 20 min at 4 °C, permeabilized using Leucoperm (AbD Serotec) and stained for intracellular cytokines with labelled mAbs for IL-17, INFγ, TNFα, GM-CSF and IL-10. The cells were fixed with 0.5% p-formaldehyde in PBS and analysed. FACS was performed using a BD-LSR Fortessa X20 (BD Biosciences) and cell populations were gated and assessed using FacsDiva 6.0 software (BD Bioscience).

### Cytokine Measurements

For quantification of cytokines, multiplex MSD kits or sandwich ELISA was used. Conditions for cytokine measurements including determining optimal dilutions of different clinical samples were carried out prior to the measurements as reported [12]. Level of the following cytokines/chemokines was determined in culture supernatants of ex vivo stimulated T and B cells and monocytes and in plasma: TNFα, IL-17, IL-1β, IL-2, IL-4, IL-6, IL-8, IL-10, IL-12p70, IL-13, IL-20, IL-22, IL-23p40, GM-CSF, IFNγ and MCP-1.

### Statistical Analyses

Comparison of patterns of cytokine production was with the Multi Experiment Viewer software (Dana Faber Cancer Institute, Boston, MA). Hierarchal clustering was with Pearson’s correlation with complete linkage. Where indicated, statistical analyses were carried out using the GraphPad Prism 6 software. The Mann-Whitney *U* test, Wilcoxon matched-pairs, signed rank test, Fisher’s exact test or the Chi^2^ test were used for the analysis of differences between or within groups, as appropriate. Positive and negative predictive values were calculated using the online program https://www.medcalc.net/tests/diagnostic_test.php.

## Results

### Patient Response to Therapy

Ninety-seven RA patients prescribed anti-TNFα were recruited to the study (Table 2) and their clinical samples used in multiple experiments described in this report. The patients were treated with one of the following four anti-TNFα agents: adalimumab, certolizumab pegol, golimumab or etanercept in combination with methotrexate. No notable or consistent differences in cytokine production by enriched cells were seen between patients receiving different anti-TNFα agents. Assessment of patients’ response to anti-TNFα was based on the EULAR response criteria at 3 months after treatment. Seventy-six patients (78%) responded to the treatment, slightly higher than previously reported [6–8, 11].

**Table 2:** Demographic and clinical data on patients included in the study

	Total	Responders (%)	Non-responders (%)	*P* value
Number of patients	97	76 (78)	21 (22)	
Age (years)	55.7	55.8 ± 13.9	57.0 ± 14.1	0.49
Gender (female:male)	2.7	2.2	6	0.22
DAS-28 before treatment	5.76	5.70 ± 0.8	5.96 ± 0.6	0.12
DAS-28 after 3 months of treatment		3.5 ± 1.2	5.8 ± 0.7	<0.01

Statistical analyses were carried out using the Mann-Whitney *U* test except for gender ratios where Chi^2^ test was used. Values provided as the mean ± standard deviation (SD).

### Distinct Pro-inflammatory T Cell Cytokine Profiles Predict Responsiveness or Lack of Responsiveness to Treatment with Anti-TNFα

In a previous study, we determined that prior to treatment with anti-TNFα, non-responder RA patients had high frequencies of IL-17^+^ T cells and, when their T cells stimulated in vitro, the cells produced significantly higher levels of IL-17 than responder patients [12]. The current study compared the profile of cytokines produced by enriched T and B cells and monocytes in responder and non-responder patients prior to and after the start of treatment to establish whether distinct inflammatory pathways associate with the disease in responder as compared with non-responder patients. Levels of TNFα, IL-17, IL-1β, IL-2, IL-4, IL-6, IL-8, IL-10, IL-12p70, IL-13, IL-20, IL-22, IL-23p19, GM-CSF, IFNγ and MCP-1 either in culture supernatants of the stimulated T and B cells and monocytes or detected in plasma collected from most of the patients at the time of sampling were measured. For clarity of our observations, only data for cytokine measurements that consistently showed significant differences between responder and non-responder patients are presented and discussed in detail in this report.

**Fig. 1:**
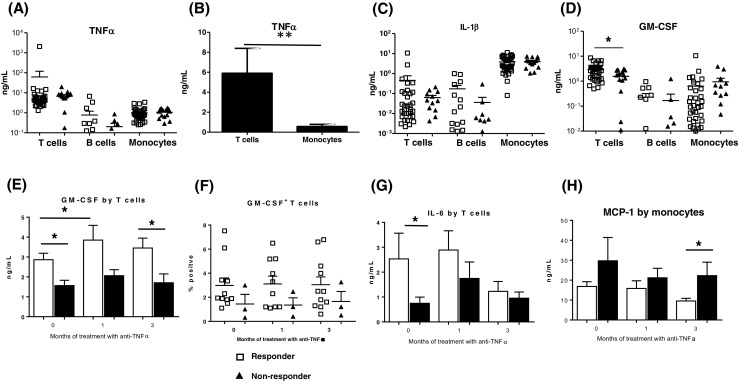
Cytokine production by T cells, B cells and monocytes in rheumatoid arthritis patients prior to and after treatment with anti-TNFα. T cells, B cells and monocytes were enriched by negative selection from the blood of RA patients immediately before treatment with anti-TNFα agents and then after 1 and 3 months. The cells were stimulated for 48 h and the level of cytokines produced determined using MSD multiplex kits. Levels of 16 cytokines were determined but only results of cytokines with notable differences are presented: **a, b** Data on TNFα; **c** IL-1β and **d** GM-CSF. **a** TNFα produced by T cells stimulated with anti-CD3/anti-CD28 mAbs, B cells stimulated with anti-IgM/anti-CD40 and monocytes stimulated with LPS. **b** Mean ± standard error of the mean (SEM) for TNFα produced by T cells and monocytes from 3 healthy controls and stimulated for 48 h with PMA and ionomycin. **c**, **d** Levels of IL-1β and GM-CSF produced by T and B cells and monocytes activated and cultured as described in **a. e** Data on GM-CSF production by T cells as described for **a** before treatment (0 months) and after 1 and then 3 months. **f** Data on the frequency of GM-CSF^+^ T cells before treatment and after 1 and 3 months in responder and non-responder RA patients. Enriched T cells were stimulated with PMA/ionomycin for 16 h and stained for intracellular GM-CSF. **g** IL-6 production by T cells and **h** CCL2 production by monocytes. The data before and after treatment are for 67 patients. Differences between responders and non-responders were assessed using two-tailed Mann Whitney *U* test. Difference at different time points in the same group was assessed using Wilcoxon matched-pairs signed rank test. *Single asterisk* indicates *P* < 0.05; *double asterisk* indicates *P* < 0.01

Monocytes are generally believed to be a main source of TNFα in joints of RA patients although there is evidence that the cytokine is also produced by T cells [29–32]. The current study confirmed that monocytes, T and B cells all produce TNFα when stimulated in vitro (Fig. 1a). However, T cells stimulated with anti-CD3/CD28 mAbs produced almost 45-fold the level of TNFα produced by LPS-stimulated monocytes (*P* < 0.0001; Fig. 1a and Table 3). To exclude the possibility that the higher levels of TNFα produced by T cells were merely due to the activation protocol and not capacity of the cells themselves, both lineage cells were independently stimulated with PMA/ionomycin in parallel experiments. The mean and SEM level of TNFα produced by T cells stimulated with PMA/ionomycin was 5.9 ± 4.3 ng/mL, >10-fold higher than what monocytes produced with PMA/ionomycin at 0.5.8 ± 0.4 ng/mL (*P* < 0.05; Fig. 1b). Before treatment, T cells from responder patients stimulated with anti-CD3/CD28 mAbs tended to produce more TNFα than non-responder patients (*P* = 0.20; Fig. 1a and Table 3).

**Table 3:** Pro-inflammatory cytokine levels produced by ex vivo enriched T cells, B cells and monocytes from patients with RA prior to the start of treatment with anti-TNFα

	T cells	*P* value	B cells	*P* value	Monocytes	*P* value
TNFα	*45.2 ± 281.5*		*0.6 ± 1.4*		*1.0 ± 0.7*	
Responders	62.3 ± 341.6	0.2	0.8 ± 1.8	0.78	1.0 ± 0.8	0.39
Non-responders	6.6 ± 5.0		0.2 ± 0.3		1.0 ± 0.5	
IL-1β	*0.31 ± 1.5*		*0.1 ± 0.3*		*3.9 ± 2.6*	
Responders	0.4 ± 1.8	0.7	0.2 ± 0.3	0.51	4.0 ± 3.0	0.83
Non-responders	0.1 ± 0.1		0.04 ± 0.1		3.9 ± 2.0	
IL-6	*1.7 ± 4.6*		*1.7 ± 3.6*		*23.7 ± 16.8*	
Responders	2.5 ± 6.2	0.27	2.5 ± 4.5	0.53	22.8 ± 16.8	0.46
Non-responders	0.8 ± 1.0		0.5 ± 1.0		26.0 ± 17.1	
GM-CSF	*2.5 ± 1.8*		*0.2 ± 0.3*		*0.8 ± 1.7*	
Responders	2.9 ± 1.9	0.02	0.2 ± 0.3	0.55	0.6 ± 1.8	0.06
Non-responders	1.6 ± 1.1		0.2 ± 0.4		1.1 ± 1.4	
CCL2					**20.1 ± 23.0**	
Responders					16.9 ± 13.8	0.45
Non-responders					29.7 ± 38.8	

Cytokine levels are presented as mean ± SD in nanograms per milliliter. For data on experiments summarised in the table cytokines produced by T and B cells and monocytes enriched from the blood of 46 responder and 21 non-responder patients are presented. Numbers in italics are for the mean ± SD of cytokines in all patients combined. *P* values <0.05 are considered statistically significant. Statistical analyses were carried out using the Mann-Whitney *U* test.

T cells from responder patients produced sevenfold higher levels of IL-1β than T cells from non-responders (Fig. 1c and Table 3). However, monocytes produced higher levels of IL-1β than T cells but there was no difference in IL-1β production by monocytes from responder and non-responder patients. As expected, B cells produced less IL-1β than monocytes but there was a trend for higher levels in responder patients (Fig. 1c and Table 3).

T cells produced significantly higher levels of GM-CSF compared with monocytes and B cells (*P* < 0.0001; Fig. 1d and Table 3). Importantly, GM-CSF levels produced by T cells were significantly higher in responder compared with non-responder patients before treatment (*P* < 0.05; Fig. 1d,e and Table 3). There was a significant increase in GM-CSF production by T cells in responder patients after 1 month of treatment but declined at 3 months (*P* < 0.05, Fig. 1e). Consistent with higher GM-CSF levels produced by T cells from responder patients, there were also more GM-CSF^+^ T cells in the blood of responder patients before (3.0 ± 2.0 vs. 1.4 ± 1.4%) and after 1 month (3.1 ± 2.0 vs. 1.4 ± 1.0%) of treatment (Fig. 1f).

Monocytes, T and B cells produced IL-6 with monocytes producing 10-fold higher levels than T and B cells (*P* < 0.0001; Table 3). T cells from responder patients produced >3-fold IL-6 levels more than T cells from non-responder patients before treatment (*P* = 0.27, Fig. 1g and Table 3).

MCP1 was produced almost exclusively by monocytes with 1.8-fold higher levels in non-responders compared with responders before and after 1 month of treatment. The difference increased to 2.3-fold after 3 months of treatment (*P* < 0.05, Fig. 1h and Table 3).

**Fig. 2:**
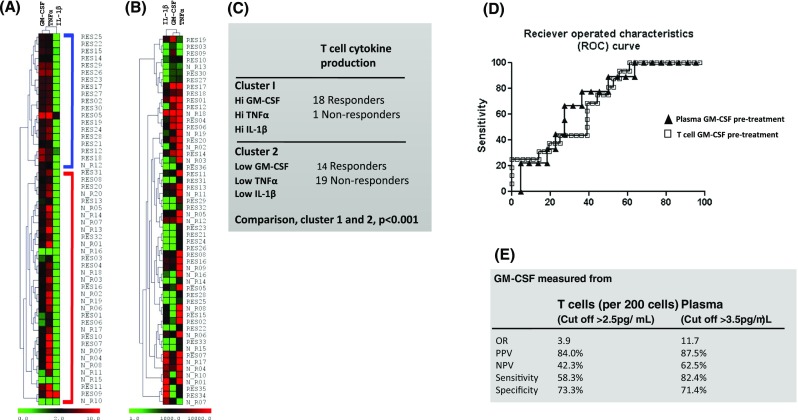
Cluster and receiver operating characteristic (ROC) analyses for predictive value of cytokines in the response to anti-TNFα. **a, b** Cluster analyses of TNFα, IL-1β and GM-CSF in RA patients pre-treatment with anti-TNFα in relation to responsiveness/non-responsiveness. Cluster analyses of GM-CSF, IL-1β and TNFα levels (**a)** by in vitro activated T cells from responder (RES) and non-responder (N_R) patients whose identity codes are indicated by numbers and (**b)** in plasma from the same patients. The cluster analyses were performed using Pearson’s correlation with complete linkage. The two clusters in **a** were highlighted with a *blue* (cluster 1) and a *red* (cluster 2) bracket. **c** Summary of data from the cluster in **a**. Significant differences between responders and non-responders were calculated using Fisher’s exact test. **d** ROC curve analyses of the response to treatment with anti-TNFα. The increasing area under the ROC curve (area under the curve, AUC) corresponds to a higher diagnostic test yield. **e** Summary of sensitivity, specificity and odds ratio (OR) for GM-CSF levels by T cells and in plasma before treatment in predicting the response to treatment with anti-TNFα. *PPV* positive predictive value, *NPV* negative predictive value. Data are presented with 95% CI

Since GM-CSF, TNFα and IL-1β produced by T cells were higher in responder patients compared with non-responder patients, we carried out a cluster analysis to assess their predictive value of responsiveness to anti-TNFα pre-treatment. A two-dimensional clustering was carried out with Pearson’s correlation and complete linkage analysis (Fig. 2a). Two clusters emerged, one containing patients with high levels of GM-CSF, IL1β and TNFα (connected with a blue bracket) and the second containing patients with low levels of the cytokines (connected with a red bracket). Eighteen of 19 patients within the first cluster (94.7%) were responders and only one was a non-responder. The second cluster contained 19 non-responders (57.6%) and 14 responders (42.4%) (Fig. 2c). The relative prevalence of responders to non-responders in each cluster was significantly different (Fisher’s exact test, *P* < 0.001). Inclusion of the other T cell-derived cytokines measured in this study did not improve the clustering and were, therefore, not included in this analysis.

### High Plasma GM-CSF Levels Confirms Association with Responsiveness to Anti-TNFα

**Fig. 3:**
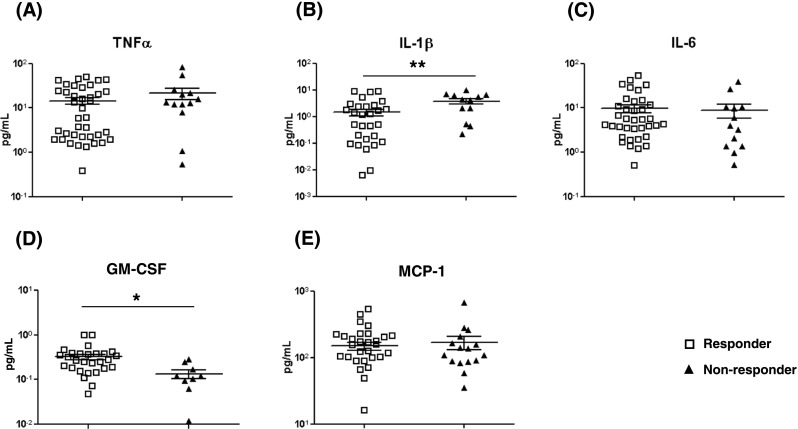
Levels of cytokines in plasma pre-treatment of RA patients with anti-TNFα. Plasma levels of cytokines were determined immediately before treatment with anti-TNFα using MSD multiplex kits. Levels of cytokines shown are for: **a** TNFα; **b** IL-1β; **c** IL-6; **d** GM-CSF and **e** MCP-1. Values are presented as the mean ± SEM in the plasma of 40 responder and 13 non-responder patients on whom plasma samples were available. Statistical analyses were with the two-tailed Mann-Whitney *U* test. *Single asterisk* indicates *P* < 0.05; *double asterisk* indicates *P* < 0.01

To explore the feasibility of developing a strategy for identifying likely responders to anti-TNFα before treatment based on the findings from the current study, we also assessed plasma levels of TNFα, IL-1β, IL-6, GM-CSF and MCP-1 pre-treatment (Figs. 2b and 3a–e). There were no differences between responder and non-responder patients in pre-treatment plasma levels of TNFα, IL-6 or MCP-1. However, high pre-treatment levels of GM-CSF were significantly associated with responsiveness to anti-TNFα (6.4 ± 4.6 pg/mL vs. 2.8 ± 2.0 pg/mL, responders vs. non-responders; *P* < 0.05; Fig. 3d). To assess the potential application of measuring GM-CSF in plasma and/or culture supernatants of T cells before treatment to predict responsiveness, we carried out a receiver operated characteristics (ROC) analysis by plotting the true positive rate (Sensitivity) as a function of the false positive rate (100-Specificity). The analyses revealed that the level of GM-CSF in culture supernatants of activated T cells gave a predictive value, area under curve (AUC), of 0.70, (0.56–0.85 at 95% confidence interval (CI); *P* < 0.05), and for plasma an AUC of 0.73 (0.53–0.90% at 95% CI; *P* = 0.09; Fig. 2d). Calculation of odds ratio (OR) showed that responder patients had an OR of 3.9 to respond to treatment at GM-CSF levels of >2.5 pg/mL produced by 200 cultured T cells and an OR of 11.7 at plasma levels of >3.5 pg/mL compared with non-responders (Fig. 2e). The predictive value of GM-CSF in culture supernatants of T cells had a specificity of 73.3% and a sensitivity of 58.3% (40.8–74.5 at 95% CI). For plasma GM-CSF, specificity was 71.4% (29.0–96.3% at 95% CI) and sensitivity 82.4% (56.6–96.2% at 95% CI). The positive predictive values, at cut-offs as stated above, for GM-CSF in culture supernatants of T cells was 84.0% (63.9 to 95.5% at 95% CI) and for plasma 87.5% (61.6 to 98.5% at 95% CI).

### The Phenotype and Activation Status of GM-CSF^+^ T Cells in RA Patients

**Fig. 4:**
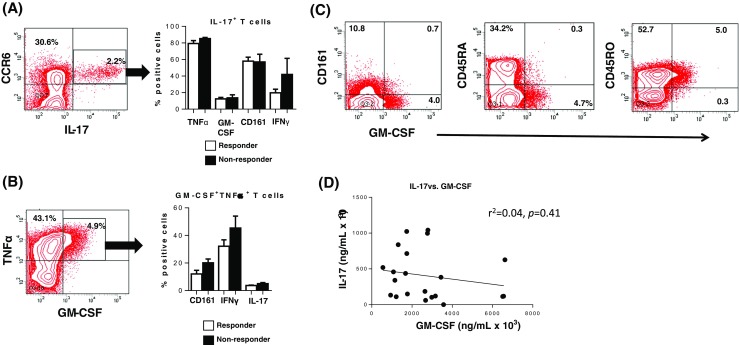
GM-CSF^+^ and IL-17^+^ T cells segregate into two different subsets. Enriched T cells were stimulated with PMA/Ionomycin for 16 h and stained for membrane proteins and intracellular cytokines as indicated. **a** Contour plot showing the percentage of CCR6^+^ and CCR6^+^IL-17^+^ in blood CD4^+^ cells and **b** TNFα^+^ and TNF^+^GM-CSF^+^ T cells in the blood of RA patients. CCR6^+^IL-17^+^ in **a** were gated and the frequency of T cells expressing intracellular GM-CSF, TNFα and IFNγ^+^ and membrane CD161 determined by FACS. **b** PMA/Ionomycin-stimulated T cells were stained for intracellular TNFα and GM-CSF and the frequency in responder and non-responder patients determined. The frequency of TNFα^+^GM-CSF^+^ T cells that co-expressed INFγ and IL-17 membrane CD161 was determined by multicolour FACS. The data represent the mean ± SEM values from seven responder and three non-responder patients. **c** Contour plots showing characteristics of the RA patients GM-CSF^+^ T cells (expression of CD45RA, CD45RO and CD161) pre-treatment. Percentages of differentially stained cell subsets are given inside the quadrants. **d** Linear regression showing the correlation between the level of IL-17 and GM-CSF produced by patients’ T cells activated with anti-CD3/anti-CD28 mAbs

There is evidence that GM-CSF can be produced by CD161^+^IFNγ^+^ ex-Th17 cells in the joint of some RA patients and by peripheral blood Th17 cells in patients with multiple sclerosis [33, 34]. Other studies, however, have shown that GM-CSF is produced by T cells other than Th17 cells [35, 36]. To assess the existence of links between GM-CSF-producing T cells and Th17 cells in our cohort of patients in relation to responsiveness to anti-TNFα, T cells were enriched, stimulated with PMA/ionomycin for 16 h, stained for membrane proteins and intracellular cytokines and analysed by FACS. We used CCR6 expression, a membrane protein expressed on the majority of IL-17^+^ T cells, to identify Th17 cells in the patients (Fig. 4a). To determine whether GM-CSF^+^ T cells overlapped with Th17 cells, we gated on CCR6^+^IL-17^+^ T cells within the enriched/stimulated CD3^+^ T cells. The FACS contour plots first confirmed that almost all of the IL-17^+^ T cells expressed CCR6 (2.2% of all CD4^+^ T cells; Fig. 4a). The gated CCR6^+^IL-17^+^ T cells were then analysed for membrane CD161 and intracellular TNFα, GM-CSF and IFNγ expression. This analysis showed that of the CCR6^+^IL-17^+^ T cells, 58.0 ± 12.7% and 57.0 ± 16.1% expressed CD161, 19.6 ± 11.7% and 41.9 ± 33.7% expressed IFNγ, 79.3 ± 9.6% and 85.3 ± 2.5% expressed TNFα but only 12.3 ± 4.8% and 13.6 ± 6.6% expressed GM-CSF in responder and non-responder patients, respectively. In contrast, analysis of GM-CSF^+^ T cells showed that almost all co-expressed TNFα^+^ (example of FACS contour plots in Fig. 4b). The GM-CSF^+^TNFα^+^ T cells (4.9% of all CD3^+^ T cells) were then gated and analysed for membrane CD161 and intracellular IFNγ and IL-17 expression (Fig. 4b). This analysis showed that of the GM-CSF^+^TNFα^+^ T cells 12.1 ± 7.0% and 20.1 ± 4.9% expressed CD161, 32.2 ± 12.2% and 45.4 ± 14.9% expressed IFNγ and 3.7 ± 1.0% and 5.0 ± 1.4% expressed IL-17 in responders and non-responders, respectively (Fig. 4b). Further analysis of GM-CSF^+^ T cells showed that they were mostly of the CD45RO^+^ effector/memory phenotype (Fig. 4c). As revealed in Fig. 4b, a relatively small proportion expressed low levels of CD161 but none expressed IL-10 (not shown) indicating that they were unlikely to be regulatory T cells (Tregs) [37, 38]. Thus, although a few Th17 cells produced GM-CSF, the majority did not (*P* < 0.0001). Likewise, only few cells within the GM-CSF^+^ TNFα^+^ T cell population produced IL-17 (*p* < 0.0001). Furthermore, when the level of IL-17 and GM-CSF from in vitro anti-CD3/CD28-activated T cell culture supernatants was compared in a subgroups of the patients, none of the patients simultaneously produced high level of both cytokines (Fig. 4d).

## Discussion

This study provides evidence that the response/lack of response of RA patients to anti-TNFα therapy is associated with differences in the frequency of T cell subsets in patients. Thus, the results show that responder patients tend to have a high frequency of T cells that produce GM-CSF in contrast to non-responders that have a high frequency of IL-17-producing T cells at the start of treatment with anti-TNFα. Furthermore, clustering analysis of the pro-inflammatory cytokines TNFα, GM-CSF and IL-1β suggests that the disease in responder and non-responder patients is likely to be driven/sustained through different inflammatory pathways. The study also suggests that T cells could be the major producers of TNFα and GM-CSF in RA patients. Consistent with the association between high frequency of GM-CSF^+^ T cells that produce high levels, the cytokine when activated in vitro and responsiveness to TNFα inhibitors is that high plasma level of GM-CSF is also detected in the patients. High level of IL-17 prior to treatment, in contrast, has an inverse relationship with responsiveness to anti-TNFα [11, 12].

About 30% of RA patients prescribed anti-TNFα do not respond adequately and a further 50% of responders relapse within 12 months of treatment [39]. Previous studies from our laboratory have revealed an association between the lack of response to anti-TNFα and high levels of IL-17-producing T cells pre-treatment [12]. These studies also showed an increase in the frequency of Th17 cells after treatment. This latter observation could be related to the fact that TNFα also has immunomodulatory functions [40]. For example, TNFα has been shown to modulate diabetes, EAE, lupus and arthritis in animal models, possibly, by inhibiting Th17 cells [41–44]. Importantly, findings from the current study show that T cells could be a major source of TNFα in vivo. This finding is consistent with studies showing that both macrophages and T cells in the synovial lining tissue produce TNFα [30, 32]. Since T cells and monocytes express TNFα receptors, the cytokine could act directly on these cells and modulate their differentiation and functions [14, 44]. Indeed, blockade of TNFα in arthritic mice led to the expansion of collagen-specific Th1 and Th17 cells and our previous studies showed that this effect is likely to be due to increased IL-12/IL-23 p40 production by monocytes and antigen-presenting cells [12, 44].

There is clearly an unmet need to identify biomarkers for evidence-based prescription of biological therapies in all RA patients. Identifying such biomarkers has been the subject of much research by many investigators. Several potential biomarkers have been reported (Table 1), but the application of such biomarkers in larger populations beyond the cohorts studied is unknown. For example, anti-cyclic citrullinated peptide antibodies and rheumatoid factors, autoantibodies traditionally associated with RA, were studied but ultimately shown to be of limited value [18, 45]. We propose that a combination of reported biomarkers, for example GM-CSF and the inflammatory protein MRP8/14 or certain micro-RNAs, could predict responsiveness to anti-TNFα in most RA patient cohorts more reproducibly [22, 26].

GM-CSF is an important cytokine for the maturation of macrophages and CD1^+^ pro-inflammatory dendritic cells [36, 46] as well as in myelopoiesis [47]. Interestingly, treatment with GM-CSF has been shown to precipitate arthritis in some cancer patients [48]. Nevertheless, although GM-CSF has well-characterised pro-inflammatory properties, it is not established if GM-CSF^+^ T cells are always pathogenic [49]. Thus, the cytokine is associated with a better prognosis in some animal models of auto-immune diseases. For example, GM-CSF has been shown to enhance the ability of dendritic cells to promote Treg cell activity and to ameliorate experimental thyroiditis and diabetes in mice [50, 51]. Although some studies have shown that GM-CSF could be co-produced by IL-17-producing T cells [33, 34], our data and other recent studies indicate that none, or only low levels, of GM-CSF are produced by Th17 cells [34, 35, 52].

Our findings also provide support for the proposition that arthritis could be driven/sustained by different inflammatory pathways in different patients resulting in a heterogeneous response to TNFα inhibitors. The lack of responsiveness to anti-TNFα in patients with low plasma levels of GM-CSF/low-frequency GM-CSF^+^ T cells indicates that disease processes in these patients are unlikely to be dominated by a pathway driven by TNFα/GM-CSF. However, an alternative explanation could be that anti-TNFα ameliorates arthritis in responder patients by binding to autoreactive effector T cells that express membrane TNFα and thereby ameliorate their pro-inflammatory effects. In non-responder patients, in contrast, it is likely that IL-1β and IL-23 produced by monocytes, when TNFα is blocked, drive Th17 cell differentiation and/or survival and promote chronic inflammation through a different pathway [12].

The current study has also revealed that monocytes from non-responder patients produce higher MCP-1 levels compared with responder patients. A significant difference was seen after 3 months of treatment, which may be accounted for by the possibility that monocytes in anti-TNFα non-responders are resistant to apoptosis [53]. However, further studies of monocytes in responder and non-responder RA patients could elucidate mechanisms that drive inflammation in patient subsets. Such studies may also reveal if there is a relationship between high IL-17 levels in non-responder patients and high-level MCP-1 production by monocytes [54]. All in all, our study is consistent with other studies published in the current issue to indicate that assessment of immune cell subsets could identify biomarkers that help in designing evidence-based strategies for prescribing the most effective biological therapies in autoimmune diseases [55, 56].

## Conclusions

This study shows that different T cell subsets are likely involved in driving arthritis in different RA patients and that depending on which T cell subset predominates, determines which patient responds to anti-TNFα. In addition to its potential clinical relevance, the study provides insights into distinct inflammatory pathways that could drive the disease and help in future patient stratification for treatment. Further in-depth studies of GM-CSF^+^TNFα^+^ T cells and IL-17-producing T cells, monocyte subsets, their proteome, transcriptome and genotype could provide invaluable new markers for targeted therapies in RA.
